# Avoidant/restrictive food intake disorder (ARFID) in New Zealand and Australia: a scoping review

**DOI:** 10.1186/s40337-023-00922-9

**Published:** 2023-11-06

**Authors:** Hannah L. Kennedy, Leonie M. Hitchman, Michaela A. Pettie, Cynthia M. Bulik, Jennifer Jordan

**Affiliations:** 1 Department of Psychological Medicine, University of Otago - Christchurch, Christchurch, New Zealand; 2 Department of Pathology and Biomedical Science, University of Otago - Christchurch, Christchurch, New Zealand; 3 Department of Psychiatry, University of North Carolina at Chapel Hill, Chapel Hill, USA; 4 Department of Nutrition, University of North Carolina at Chapel Hill, Chapel Hill, USA; 5 Department Medical Epidemiology and Biostatistics, Karolinska Institutet, Stockholm, Sweden; 6 Specialist Mental Health Clinical Research Unit, Te Whatu Ora, Waitaha, Christchurch, New Zealand

**Keywords:** Eating disorders, Fussy eating, Picky eating, Scoping review, Australia, New Zealand

## Abstract

**Background:**

Avoidant/restrictive food intake disorder (ARFID) is an eating disorder that involves restrictive or avoidant eating behaviour not related to weight or body image concerns. It was first included in the Diagnostic and Statistical Manual of Mental Disorders–fifth edition (DSM-5) in 2013. ARFID frequently begins in childhood and can have serious psychosocial impacts and detrimental health consequences when nutritional and energy needs are persistently unmet. This systematic scoping review focuses on Australasia, synthesizing the current literature landscape on ARFID, and offering recommendations for targeted, actionable research directions for both funders and researchers.

**Methods:**

Online databases and university thesis repositories were systematically searched for studies examining ARFID in the New Zealand or Australian population since 2013. Database search results were exported to Rayyan software, and two independent reviewers screened all identified sources, prior to extraction of key data.

**Results:**

Twenty-nine studies and one thesis from 138 screened sources were eligible for inclusion. Frequent study types were treatment interventions and cross-sectional studies, with populations including individuals with ARFID, ED service populations, parents/caregivers, health professionals, and non-clinical populations. ARFID presents in a range of settings and is associated with poorer quality of life and significant functional impairment. Assessment of ARFID was varied, and no specific treatment guidelines for ARFID have been written as yet.

**Conclusion:**

This review calls for more accurate prevalence estimates of ARFID in children and larger-scale studies in all ages using validated measures. It emphasizes the need for education and training of healthcare professionals, and interdisciplinary collaboration. Established interventions like behaviour analytics should be considered, and more comprehensive research is needed on interventions for ARFID, including controlled trials and longitudinal studies. Urgent research is needed to improve outcomes for those affected by ARFID.

## Abstract (plain language summary)

This scoping review examines all published literature on Avoidant/Restrictive Food Intake Disorder (ARFID) in the Australasian region since the disorder was first recognized in 2013. ARFID is an eating disorder marked by restrictive or avoidant eating behaviour unrelated to weight or body image concerns. The disorder can have serious psychosocial impacts and detrimental health consequences when nutritional and energy needs are persistently unmet. The review identifies the methods, participants, and key findings of the studies on ARFID and suggests targeted and actionable research goals for researchers and funders. It calls for more accurate information on how common ARFID is in children, for larger-scale studies using validated measures, and emphasizes the need for education and training of healthcare professionals, and a collaborative approach to treatment. We also underscore the need for longitudinal studies to better understand the landscape of ARFID in Australasia.

## Introduction

Avoidant/restrictive food intake disorder (ARFID) is an eating disorder diagnosis first included in the Diagnostic and Statistical Manual of Mental Disorders–fifth edition (DSM-5); Feeding and Eating Disorders section in 2013 [1] It has more recently been included in the International Classification of Diseases 11th version (ICD-11) [2], which was released in 2019 and in effect from 2022. The presentation of ARFID is characterised by food avoidance or restriction due reasons such as to low appetite/food apathy, sensory sensitivity, or fear of undesired consequences (e.g., choking). This restriction occurs without the weight and body image concerns associated with anorexia nervosa. Particularly in children, these behaviours were historically described as picky or fussy eating, although the severity of ARFID extends well beyond developmentally typical picky eating. In ARFID, nutritional and energy needs are often persistently unmet resulting in growth compromise, weight loss, malnutrition, and in severe cases dependence on nutritional supplementation or feeding tubes. Psychosocial impairment may also arise, impacting on an individual’s ability to navigate daily activities such as school, or family mealtimes.

The prevalence of ARFID is currently unknown, but estimated to be between 0.5 and 5% in children [3–6] and adults [7–10]. Onset of ARFID can occur at any stage through the lifespan [11, 12], although onset is typically in childhood or adolescence [13–15]. ARFID appears to occur at similar frequencies in females and males [3, 4, 9, 10], in contrast to many other eating disorders that display a female predominance. It is unclear if ARFID precipitates migration to another eating disorder such as anorexia nervosa as previously suggested [16], or in what percentage of individuals ARFID may persist long-term [17]. No evidence-based treatment guidelines are as yet available, although several groups are actively evaluating efficacy of new treatment approaches [18–20]. ARFID adaptions of Family Based Therapy (FBT)[21–23], and Cognitive Behavioural therapy for ARFID (CBT-AR) [18, 24] have both demonstrated preliminary efficacy. Little long-term data is available to inform management practice.

Historically, ARFID symptomology has been captured by multiple diagnostic classifications, and terms. These include DSM classifications of Feeding Disorder of Infancy or Early Childhood, and EDNOS, and ICD classification of Feeding Disorders of Infancy and Childhood in previous editions of both manuals. Although not an official classification, the term Paediatric Feeding Disorder (PFD) is widely used to describe a broader range of feeding difficulties that can have medical, developmental, or behavioural causes. Although ARFID would come under the umbrella of PFD, many of those other disorders within PFD encompass factors like age-inappropriate feeding skills or medical dysfunction impacting the eating process, which, if present, would exclude an ARFID diagnosis. There is considerable diagnostic overlap between PFD and ARFID, and different healthcare specialties and professionals may have varying levels of familiarity with the ARFID diagnosis. For example, gastroenterologists may be more likely to identify PFD, whereas mental health professionals may be more familiar with ARFID. This inconsistency in diagnosis highlights the need for greater clarity and awareness among healthcare providers [25] about the differences between PFD and ARFID.

The relatively recent inclusion of ARFID in diagnostic manuals (DSM-5 and ICD-11), as well as inconsistencies in ARFID characterization among various specialities and healthcare professionals, may have also contributed to the under-representation in academic and clinical literature. Many studies may not have accurately captured ARFID cases, and as a result, the prevalence of ARFID may be underestimated. Moreover, local research is essential to assess the generalizability of international findings, while considering the unique sociocultural context and Indigenous participants.

An existing international systematic review on ARFID is available [13] that provides a valuable summary of the body of evidence through to April 2019. Limited data are available for Australasia in this time period, however, with multiple studies published in this region after 2019. Bourne’s 2020 review captured only three studies from Australia and none from New Zealand (NZ). A scoping review of academic sources (including peer-reviewed and theses) is needed to provide up to date information on ARFID research so we can consider the findings within a unique context, accounting for local perspectives and experiences (including those of the Indigenous people) of both countries.

This present review focuses on NZ and Australia literature, incorporating new studies published beyond the timeframe of previous reviews (as well as theses which were not included in previous search methods), providing a current and comprehensive overview of ARFID within Australasia. This is intended as a resource for Australasian researchers planning future research in ARFID, to better understand what data are currently available, and where significant gaps exist that should be addressed to best promote advancing knowledge in this arena. This is a timely synthesis given that the Medical Research Future Fund (MRFF) set up by the Australian Government in 2015 as a long-term investment in Australian health and medical research grew to $20 billion in 2020, and organisations such as Whāraurau in NZ are currently leading ARFID initiatives.This study provides important local data for the benefit of funders, service providers, and support groups of those with ARFID and their families.

### Objectives

1. Provide a comprehensive and current synthesis of all available evidence on ARFID within the Australasian context
2. Identify key concepts, relevant target groups, and gaps in the literature
3. Identify areas of focus for NZ and Australian funding bodies

## Methods

This review was developed around the research question “To date, what parties of interest (or ARFID population foci), methodologies, and results have been reported by studies examining ARFID within Australasia?” These methods were developed in accordance to the Preferred Reporting Items for Systematic Reviews and Meta-Analyses (PRISMA) extension for scoping reviews [26].

### Protocol and registration

This systematic review and method protocol were registered in the OSF Registries October 12, 2022 (DOI: https://doi.org/10.17605/OSF.IO/HFMXE). No major deviations from the protocol occurred, minor deviations are listed below:

- The term “stakeholders” was replaced with “parties of interest (or ARFID population foci)” as this better reflected the authors intention to include groups such as healthcare providers and caregivers of ARFID patients, as well as those directly impacted by ARFID.
- Search terms were limited by database fields in certain instances (described below) rather than searching in “all fields” as per the registered protocol where this resulted in a large number of non-specific results.

### Eligibility criteria

The concept of interest is studies that include and describe all aspects of ARFID. The geographical location of the study must be within NZ and/or Australia and the date of publication on or after 1 June 2013 as this relates to the formalisation of ARFID as a diagnosis in the DSM-5. Accepted types of sources include any study design, practice guidelines, studies published in peer-reviewed journals, and articles published in English language. Review articles (including meta-analysis, systematic and scoping reviews), conference abstracts, commentaries, or poster presentations without an associated full-text article in academic peer reviewed literature, and book chapters in which no primary data or research are detailed are excluded. Participants of all ages who have a reported diagnosis of ARFID (with or without co-morbid conditions), or who meet DSM-5 or ICD-11 criteria for ARFID, or have been assessed by clinicians to have ARFID using psychometric or structured measures are included. Additional parties of interest, such as healthcare providers or caregivers of ARFID patients, are also included. Excluded are participants where behaviour is attributable to another eating disorder or exclusively explained by an existing medical condition.

### Information sources

Searches were conducted across the following databases: Ovid (APA PsycInfo, APA PsycExtra, and EMBASE), PubMed and Web of Science. The Australian New Zealand Clinical Trials Registry (ANZCTR) and grey literature (Australia and New Zealand University thesis repositories) were also searched.

### Search

Searches were performed between the 12^th^ of Oct 2022 and the 22^nd^ Feb 2023.

An initial database search (Web of Science) took place in August 2022 to identify initial guide search terms and determine the feasibility of scoping search. The initial search supported use of the terms with no field restrictions (such as title or abstract). Location was more broadly limited to Australasia by combining previous search terms with “New Zealand”, “NZ”, “Australia”, or “Australasia” (all fields, combined with OR), rather than restricting to only the location search field which did not always reflect study location.

Refined search terms are as follows:

Web of Science and PubMed were searched using the terms "ARFID" OR “Avoidant/restrictive food intake disorder” AND “NZ” OR “New Zealand” OR “Australia” OR “Australasia” with no field limits or restrictions.

OVID (including PsycInfo, PsycExtra, and EMBASE) was searched using "ARFID" or "Avoidant restrictive food intake disorder" (limited only to the abstract field) AND "New Zealand" or "NZ" or "Australia" or "Australasia" with no field limits or restrictions. In this case initial searching of all fields for all keywords returned a large number of unrelated studies, which were reduced by employing the abstract field limit in the database search parameters.

Following screening of records from OVID, Web of Science, and PubMed database searches, two authors were noted to be prominent in included studies, Phillipa Hay and Tessa Taylor. Publications by these key authors were specifically searched for in Web of Science using the search terms “Author = (Taylor, Tessa* OR Taylor, T*) AND ALL = (feeding OR eating OR food intake)”, “Author = (Hay, Phillipa* OR Hay, P*) AND ALL = (feeding OR eating OR food intake)”. Due to the scarcity of ARFID literature relating to New Zealand, the only two authors who included NZ participants in the screened literature were also included in the key author search in Web of Science using the search terms “Author = (Jackson, B* OR Jackson, Bianca*) AND ALL = (feeding OR eating OR food intake)”, “Martin Sellbom – Author = (Sellbom, M* OR Sellbom, Martin*) AND ALL = (feeding OR eating OR food intake)”.

To locate and identify relevant NZ masters and doctoral theses as part of the grey literature search strategy, institutional research archives were searched using the terms “ARFID” and “Avoidant/restrictive food intake disorder” with no filters. The University of Otago (OURArchive), University of Waikato (Research Commons), Massey University (Massey Research Online), Victoria University (ResearchArchive), University of Canterbury (research repository) and the University of Auckland (ResearchSpace). A total of 5 sources were identified from the University or Canterbury, and the University of Auckland. Access to results from the University of Auckland were restricted and retrieval of these theses required personal communication with authors and library interloan requests.

Australian theses were searched for using Trove National Library of Australia, which connects to hundreds of Australian libraries including University libraries. No relevant theses were identified through this resource using the search terms “ARFID” and “Avoidant/restrictive food intake disorder”.

Personal communication with authors identified one further thesis that was assessed for inclusion.

Hand curation of citations (reviewing the reference list) within key papers did not identify any further relevant sources of evidence.

### Selection of sources of evidence

Results from all searches were uploaded to Rayyan systematic review software (available at https://www.rayyan.ai/). Deduplication and subsequent record screening was performed in Rayyan by two independent reviewers (HK and LH). Initial screening included a review of the title and abstract of each record for relevance. Full text versions of records that were retained after this screening were retrieved where possible, and a full text review was performed in the same manner as the initial screen. Three inconsistent decisions between reviewers were discussed, and further assessed by a third reviewer (JJ).

### Data charting process/ data items

A data-charting table was developed to determine which variables to extract from evidence sources. This included relevant study population(s) of interest, health discipline(s) of authors, study methodologies, and key data instrument or measures used. The focus of each study, alongside the key finding were summarised. Demographic data are reported where available for age, gender, and ethnicity. Age range and gender data (%) were presented where available. Ethnicity data was presented as reported, except where terms such as ‘New Zealand European’ and ‘Caucasian’ were presented as ‘European’ for consistency.

### Synthesis of results

Studies were grouped according to the primary methodology used. Data were summarized by the type of population, demographics, settings, key data collected (including measures used), and relevant findings.

## Results

### Selection of sources

**Fig. 1:**
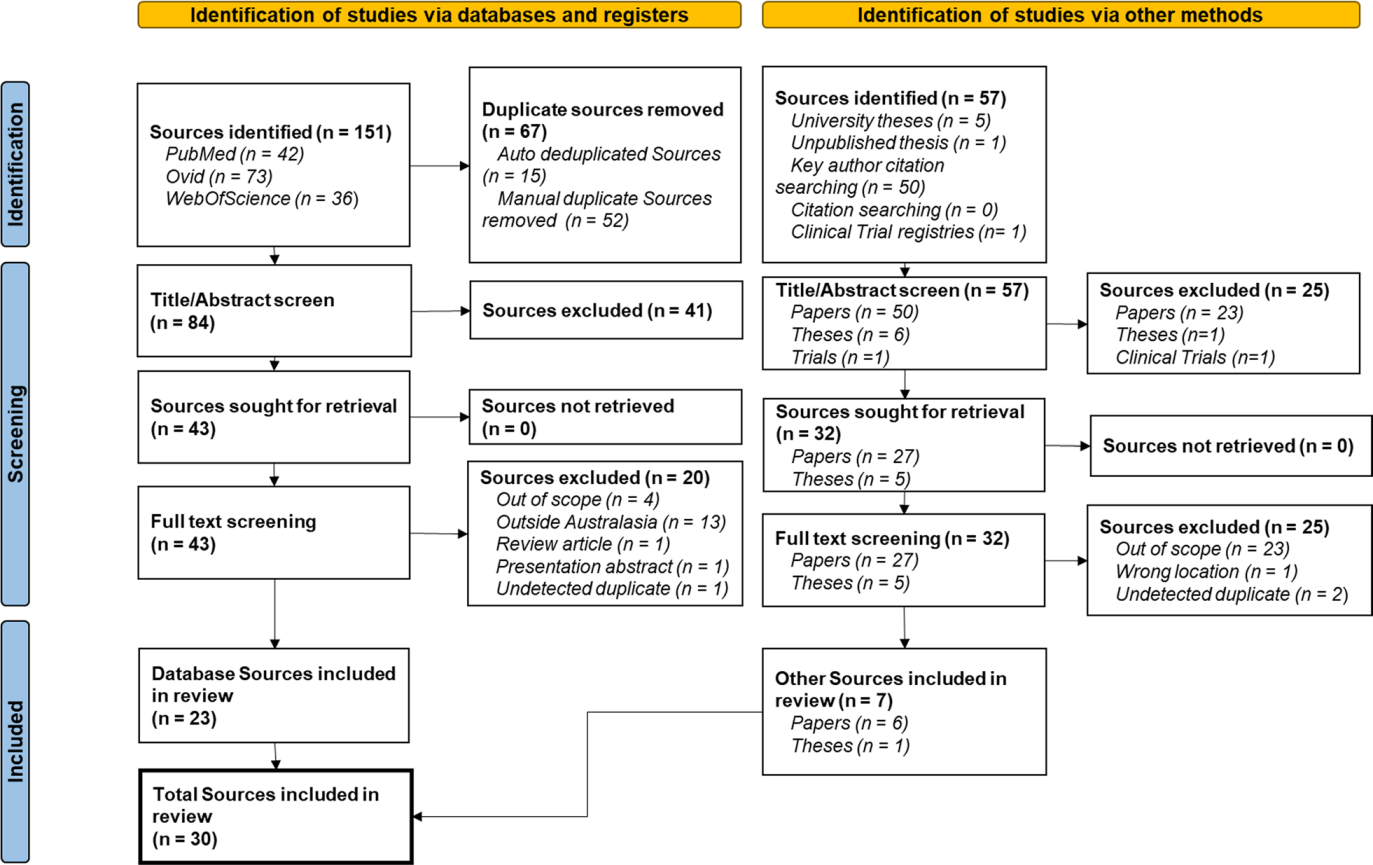
Prisma flow diagram detailing identification, screening and inclusion/exclusion of sources in this review. Italicized text represents a breakdown of the total sources described in each box

A total of 141 unique sources were initially identified for screening from database searches, and grey literature. Following the PRISMA guidelines, Fig. 1 details the number of sources excluded at each stage and the associated reasons. Of the 75 sources assessed for eligibility, reasons for exclusion were; out of scope (n = 27), outside Australasia (n = 14), a review article (n = 1), presentation abstract (n = 1), and previously undetected duplicates (n = 3). A total of 30 sources were accepted for inclusion in this scoping review.

### Characteristics of sources

#### Methodologies

Included studies were grouped by methodology, including case reports (4 records, Table 1), treatment interventions (15 records, Table 2), cross-sectional (9 records, Table 3), and production of guidelines (2 records, Table 4).

#### Journal audience

Sources in this study were published in journals with a range of intended audiences. A focus on eating disorders (5 studies [27–31]), psychiatry (4 studies [32–35]), and paediatrics (4 studies [36–39]) commonly featured. In addition, journals relating to adolescence [40]; gastroenterology [41]; learning and motivation [42]; behaviour change [43–46]; speech, language, and hearing [47]; disability [48]; and autism and developmental disorders [49] were used to disseminate ARFID research among others, with the journal often being closely relating to the setting in which the sample was investigated.

#### Study population focus

Study groups included individuals with ARFID (usually with comorbid Autism Spectrum Disorder (ASD) or anxiety), ED service populations, adult ED inpatients [41], parents/caregivers of children with ARFID [50, 51], healthcare professionals [47, 52], a sample of young autistic adults, and non-clinical population samples. Two secondary analyses used data from the South Australian Health Omnibus Surveys [7, 34].

Of the guidelines, one was directed at EDs in general [53], whereas the other focussed specifically on EDs in people of higher weight [30].

#### Setting

Case studies included presentations to medical services (emergency, paediatrics, multidisciplinary team) for ARFID-associated medical conditions, or to eating disorder services, or psychiatry services. The most common setting (due to a single prolific author) was in-home paediatric feeding treatments (11 studies).

Cross sectional studies were undertaken in psychiatric and epidemiological settings. Two surveys of healthcare practitioners focussed on mental healthcare professionals [52], speech language therapists, dietitians, paediatricians, and general practitioners [47].

#### Data collected

Data frequently collected in studies including ARFID participants were physical measures (weight, height, BMI), biological measures, (including nutritional blood screens [28, 39]), background history, and a range of mental status examinations (including questions based on the DSM-5 Eating Disorders assessment). Use of Magnetic Resonance Imaging (MRI) was reported in several case studies [28, 36, 54]. For treatment intervention studies, observational measures of food consumption and mealtime behaviours as well as social/caregiver acceptability ratings were frequently recorded. Among studies that surveyed healthcare professionals, additional information such as, what role, years of experience, and geographical region the respondent worked in was collected.

Cross sectional studies predominantly favoured questionnaire instruments. Commonly used or adapted were the Health Related Quality of Life – short-form 12 v1 (2 studies [7, 34, 35]) and the Eating Disorders Examination (EDE [34, 35, 54]/EDE-Q [27]). There was no single preferred measure of ARFID, however, one frequently repeated brief ARFID assessment was ‘Are you currently avoiding or restricting eating any foods to the degree that you have lost a lot of weight and/or become lacking in nutrition (e.g. have low iron) and/or had problems with family, friends or at work?’ [7, 34, 35]. This assessment was used in the Health Omnibus surveys [55], and is therefore reported by studies that utilised these data. Two studies presented healthcare professionals with clinical vignettes and asked corresponding questions [47, 52].

### Sample characteristics

Study population sizes ranged from n = 1 in case reports, n = 1–32 in treatment interventions, and n = 10–6156 in cross sectional studies. No large cross-sectional studies have been undertaken yet on a purely clinical ARFID population in Australasia. The ages reported in case reports ranged from 11 to 64 years (4 studies), with treatment intervention studies typically performed on younger individuals (age range 2–13 years; 12 studies). Two cross sectional studies that surveyed healthcare professionals had larger sample numbers with 66 and 141 participants respectively [47, 52].

Larger sample sizes were present for studies that assessed representative populations (4 studies) (n range = 2732–5609) although the total reported number of participants with ARFID in these studies was modest; n = 9 (2014) [7], n = 10 (2015) [7], n = 13 [35]. Only two studies [29, 56] used a representative child sample (age range 5–12 years), whereas the other representative studies reflected the adult population with reported mean ages > 35 years.

Ethnicity data were commonly unreported (14 studies), with European ancestry the most common, and additional ethnicities often listed without frequency data. An exception to this was treatment intervention studies where ethnicity was reported in 11 of the 15 studies.

Two [56, 57] of the three studies undertaken in NZ reported NZ Māori and/or Pacific Island participants. Only two Australian sources reported First Australian participants [34], and Pacific Island participants [51] respectively.

### Results of individual sources of evidence

See Tables 1, 2, 3 and 4.

**Table 1:** Case reports

Author (year) Country Journal	Population focus	Study Focus	Setting	Study type	Key data	n	Gender (Age)	Ethnicity	Summary results
*Chandran NSW, Australia *[28] *Int J Eat Disord*	Clinical case, ARFID	Subacute combined degeneration of spinal cord secondary to B12 deficiency	Emergency, gastroenterology, Adolescent Medicine	Case description	Biological (nutritional blood screen, MRI; CT scan; DEXA bone scan; body fat measure); background history; neurological status; mental status; and neuropsychology examinations	1	M (17y 9 m)	Not stated	Delayed ARFID diagnosis due to late presentation. Transitioned from nasogastric tube to varied oral diet during inpatient treatment period with multidisciplinary team
*Sanders * *(NSW, Australia)* [54] J Clin Neurosci	Clinical case, ARFID	Presented with behavioural and cognitive abnormalities. ED history (including retrospective diagnosis of ARFID) with subsequent detection of C9orf72 expansion and diagnosis of familial frontotemporal dementia (FTD)	Neurology clinic	Case description	Background history; physical examination (including BMI); Montréal Cognitive Assessment; Neurological examination; MRI; DSM-5 ED assessment	1	F (64y)	Not stated	Unusual primary behavioural manifestation of FTD exhibited as ARFID with low weight and features of subthreshold AN
*Mina* *(WA, Australia)* [36] J Paediatr Child Health	Clinical case, ARFID	Presentation and treatment for adolescent male with nutritional optic neuropathy secondary to ARFID	Emergency, General Paediatrics	Case description	Background history; x-ray; WISC-V; dietary history; physical examination (anthropometric measures, MRI, bone mineral density scan); vision assessments (visual acuity, eye movement, retinal examination); biological measurements (haemoglobin, folate, vitamin D and vitamin A)	1	M (15y)	Not stated	Patient's dietary history was critical in establishing diagnosis of nutritional optic neuropathy secondary to ARFID
*Mahoney (QLD, Australia)* [39] J Paediatr Child Health	Clinical case, ARFID, anxiety	Vitamin A deficiency in ARFID	Paediatrics	Case description	Nutritional screening panel; ophthalmology review; physical examination; case history	1	F (11y)	Not stated	Diagnosis of vitamin A deficiency retinopathy with undetectable levels of vitamin A and B12 and selenium deficiency. ARFID diagnosis with comorbid generalised anxiety. One month of enteral nutrition resulted in normalised vitamin levels and normalisation of retinopathy changes. Intensive therapy with multidisciplinary team required to address food-related anxiety prior to discharge

Abbreviations: Avoidant/Restrictive Food Intake Disorder (ARFID), Body mass index (BMI), Computerised Tomography (CT), Dual X-ray Absorptiometry (DEXA), Eating disorder (ED), Familial Frontotemporal Dementia (FTD), Female (F), Journal of Clinical Neuroscience (J Clin Neurosci), Journal of Paediatrics and Child Health (J Paediatr Child Health), Magnetic Resonance Imaging (MRI), Male (M), The Diagnostic and Statistical Manual of Mental Disorders, Fifth Edition (DSM-5), The International Journal of Eating Disorders (Int J Eat Disord), Wechsler Intelligence Scale for Children, Fifth Edition (WISC V)

**Table 2:** Treatment interventions

Author and year (Country)	Study Population focus	Study Focus	Setting	Methodology	Key data collected	n	Gender (Age)	Ethnicity	Summary results
Steen (SA, Australia) [27] Int J Eat Disord	Clinical Case ARFID	Presentation and treatment for adult male with co-occurring food avoidance, and alcohol use disorder	Eating disorders service (outpatient)	CBT 10 sessions	Background history; MINI; BMI; EDE-Q; DASS-21	1	M (42y)	Not stated	Increased diversity in diet, reduced binge drinking at end treatment, but relapse at three-month follow up, with increased drinking binges, decreased overall food intake, and severe depression and anxiety. Chronic alcohol use disorder inhibited effectiveness of short therapy, and definite diagnosis of ARFID
Taylor (Australia) [43] Eur J Behav Anal	Clinical case, ARFID*, ASD	Replication of a side deposit treatment package in a home setting	Paediatric feeding	Behaviour analytic treatment	Realtime participant and feeder behaviours, interobserver agreement, procedural integrity and caregiver satisfaction	1	M (9y)	European	Treatment initially unsuccessful, but with addition of side-deposit consumption increased 100%. Treatment gains maintained over a year post implementation
Taylor (Australia) [40] J Adolesc	Clinical case, ARFID	Application of established behaviour intervention procedure in a home setting	Paediatric feeding	Behaviour-analytic treatment	Measures of consumption of food, interobserver agreement, procedural integrity, social acceptability, and caregiver satisfaction	1	F (13y)	Not stated	Consumption increased from 7 foods at baseline to 61 foods at 2 weeks post treatment. Parents reported high social acceptability of intervention (4.88/5), and high caregiver satisfaction (4.82/5). At 9-month follow-up food consumption remained stable
Taylor (Australia) [48] J Dev Phys Disabil	Clinical case, ARFID, ASD	Application of established behaviour intervention procedure (exit criterion) in a home setting	Paediatric feeding	Behaviour-analytic treatment	Measures of consumption of food across food preference groups, procedural integrity, interobserver agreement, social acceptability, and caregiver satisfaction	1	M (11y)	Not stated	100% decrease in inappropriate mealtime behaviour, and 99% reduction in negative vocalisations at end of treatment evaluation. Variety of 79 foods achieved in < 2 weeks. Parents reported complete resolution of feeding problem at 2-year follow-up, high social acceptability of intervention, and high caregiver satisfaction
Taylor (Australia) [33] J Pediatr Psychol	Clinical case, ARFID*, ASD	Side-deposit treatment in home-based program	Paediatric feeding	Behaviour analytic (side deposit)	Measures of consumption of food, procedural integrity, and social acceptability)	2	M (5y) M (4y)	Asian European European	Consumption for both participants increased by 100% when the side deposit was added to the treatment package. Increased consumption was maintained up to 3 years
Taylor (Australia) [42] Learn Motiv	Clinical case, ARFID, ASD	Effectiveness of behaviour-analytic treatment in a home setting for a child with no history of chewing behaviour	Paediatric feeding	Behaviour-analytic treatment	Measures of consumption of food across food preference groups, procedural integrity, interobserver agreement, social acceptability, and caregiver satisfaction	1	M (5y)	European	Successfully introduced chewing and swallowing behaviour during treatment period. 100% increase in consumption and chewing and 100% decrease in inappropriate mealtime behaviour at end-treatment. At 1-year follow-up, parents rated progress at 4–5/5, high social acceptability of intervention (5/5), and high caregiver satisfaction (5/5)
Taylor 2020 (Australia) [44] Learn Motiv	Clinical cases, ARFID (in siblings of ARFID cases)	Intensive in-home feeding program concurrent to an older siblings’ feeding treatment	Paediatric feeding	Behaviour analytic (baseline escape, differential attention, contingent access, noncontingent access)	Realtime measures of participant and feeder behaviours (frequency of bites, time to swallow, inappropriate mealtime behaviours) and total food consumption. Interobserver agreement, and caregiver satisfaction	2	M (2.5y) M(2y)	Asian Australian South American Australian	Short (< 10 days), intensive in-home treatment resulted in increased food consumption 100%, and decreased negative mealtime behaviours for two developmentally normal toddlers. At 3-year follow-up treatment gains were maintained
Burton (Melbourne, Australia) [58] J Can Acad Child Adolesc Psychiatry	Clinical cases, ARFID, ASD	Application of Family Based Treatment (FBT) + Unified Protocols for Transdiagnostic Treatment of Emotional Disorders in Children and Adolescents (UP-C/A)	Paediatric psychology	FBT + UP-C/A	Treatment goals (no objective outcome measures used)	2	F (6y) F (11y)	Not stated	Application of FBT + UP-c/A for ARFID with comorbid ASD appeared to contribute towards increased oral intake and food variety, and reduced reliance on NGT feeding
Taylor (Australia) [45] Behav Change	Clinical case, ARFID, ASD	Effectiveness of move-on treatment component	Paediatric feeding	Behaviour-analytic treatment	Measures of consumption of food across food preference groups, procedural integrity, interobserver agreement, social acceptability, and caregiver satisfaction	1	F (5y)	Asian Australian	Move-on component added to treatment package resulted in increased consumption and decreased time to consume foods
Taylor 2022 (Australia) [46] Behav Modif	Clinical case, ARFID, ASD	Medication administration in children with feeding disorders	Paediatric feeding	Behaviour-analytic treatment	Behaviour frequency, latency duration, procedural integrity, interobserver agreement, social validity and treatment acceptability	1 1	M (5y) M (8Y)	East Asian Australian South Asian Australian	Both participants demonstrated 100% increase in medication consumption with reduced inappropriate mealtime behaviour and quicker consumption. Treatment results were rapid (within 10 min of session 1)
Taylor (Australia) [37] Infants & Young Children	Clinical case, ARFID*, ASD	Redistribution treatment (movement of food in mouth via external tool, such as infant gum brush to discourage packing food in cheeks) in a home-based program	Paediatric feeding	Behaviour analytic (move-on, baseline escape, contingent access, non-contingent access, differential attention, redistribution)	Frequency of clean mouth, acceptance, inappropriate mealtime behaviour. Duration of latency to clean mouth, negative vocalisations, latency to acceptance. Interobserver agreement, social validity, treatment acceptability	2	F (4y) M (5y)	Asian Australian European	Patient A increased from 2 foods (within 1 food group) to 70 foods at end of 3 day treatment evaluation. Patient B reached 77 foods. Consumption increased to 100% and results were maintained at 6-month follow up
Taylor 2022 (Australia) [49] J Autism Dev Disord	Clinical case, ARFID, ASD	Treatment comparison	Paediatric feeding	Multi-element single-case experiment design	Latency to clean mouth, negative vocalisations, inappropriate mealtime behaviour, and expulsion events	1	M (4y)	European	Use of a liquid chaser to treat packing behaviour significantly decreased swallowing latency was more effective than multiple other treatments, including move-on, puree chaser, brush distribution, non-removal, re-presentation, contingent access, and differential attention methods
Taylor (NSW, Australia) [38] Acta Paediatr	Clinical case, ARFID, ASD, Developmental delay, Intellectual disability	Maintenance of specialist treatment gains at home by trained parents	Paediatric feeding	Controlled consecutive case series Behaviour-analytic feeding treatment	Measures of consumption of food, procedural integrity, interobserver agreement, social acceptability, and caregiver satisfaction	26	22 M (2-13y, mean = 6y)	15 European Australian, others were of Asian, Arabic and European ethnicities	Individualised treatments were tailored to the child, and parents trained to a high standard to continue treatment protocol at home. At 2-3ys post treatment, 21 parents reported that their child’s feeding problem was better than before treatment. 5 parents reported that the feeding problem had resolved
Taylor (Australia) [50] Child Fam. Behav Ther	Caregivers of children with paediatric feeding disorders	Social validity of treatment for paediatric feeding disorders	Paediatric feeding	Retrospective analysis Social validity correlations	Interobserver agreement, procedural integrity, caregiver satisfaction and acceptability measures	32	24 M (2-13y, mean = 6y)	18 European Australian, others = Asian, Arabic, European ethnicities	No significant correlations between treatment social validity and variables such as participant characteristics and goals, treatment procedures and treatment outcomes. Longer treatment programmes were associated with higher acceptability, although social validity ratings were very high across the sample
Taylor (Australia) [51] Behav Interv	Caregivers of children with ARFID	Evaluate caregiver treatment acceptability across the range of specific procedures for paediatric feeding disorders, at pre- and post-treatment	Paediatric feeding	Caregiver survey	General Treatment Preferences survey (prior to starting program) Acceptance Procedures/ Clean mouth/Swallowing Procedures surveys (prior to component implementation). Treatment acceptability survey (items similar to the AARP and IRP-15), with open-ended survey questions (after program discharge)	6	4 F (not stated) 2 M (not stated)	Asian, Arabic, European, South American, and Pacific Island ethnicities /nationalities	Caregivers unanimously gave strong ratings of the importance of goals, and preferred that treatment be effective and quick, over minimizing side effects

Abbreviated Acceptability Rating Profile (AARP), Acta paediatrica (Acta Paediatr), Autism spectrum disorder (ASD), Avoidant/Restrictive Food Intake Disorder (ARFID), Behavior Modification (Behav. Modif.), Behaviour Change (Behav Change), Behavioural Interventions (Behav Interv), Body mass index (BMI), Child & family behaviour therapy (Child Fam. Behav Ther), Depression Anxiety and Stress Scale 21 (DASS-21), Eating Disorder examination questionnaire (EDE-Q), European journal of behavior analysis (Eur J Behav Anal), Family Based Treatment (FBT), Female (F), Intervention Rating Profile-15 (IRP-15), Journal of Adolescence (J Adolesc), Journal of Developmental and Physical Disabilities (J Dev Phys Disabil), Journal of Pediatric Psychology (J Pediatr Psychol) Journal of autism and developmental disorders (J Autism Dev Disord), Journal of the Canadian Academy of Child and Adolescent Psychiatry (J Can Acad Child Adolesc Psychiatry), Learning and Motivation (Learn Motiv), Male (M), Mini International Neuropsychiatric Interview (MINI), Nasogastric tube (NGT), The International journal of eating disorders (Int J Eat Disord), Unified Protocols for Transdiagnostic Treatment of Emotional Disorders in Children and Adolescents (UP-C/A)

^*^ Indicates that the ARFID diagnosis was not specified in the text, but confirmed with author in personal correspondence

**Table 3:** Cross-sectional studies

Author and year (Country)	Study Population focus	Study focus	Setting	Methodology	Key data collected	Sample n	Gender (Age)	Ethnicity	Summary results
*Acharya * *(NSW, Australia)* [41] J Gastroenterol Hepatol	Adult ED in-patients	Retrospectively characterise and report on adult patients admitted to inpatient refeeding in context of EDs	Multidisciplinary team (gastroenterology psychiatry specialised nursing dietetics social work)	Retrospective clinical data collation	Diagnosis, demographics, length of stay, type of refeeding (oral, nasogastric tube), BMI (admission/discharge), medical complications, Mental Health Act scheduling, and relapse and readmission rates	ARFID n = 2 Other ED n = 8	5 F (> 16y)	Not stated	All admissions had episodes of hypoglycaemia. The average change in BMI from admission to discharge was 1.7 kg/m^2^ but was -1.8 to 3.8 kg/m^2^ for patients with AN or ARFID
*Burt * *(Australia)* [34] BMC Psychiatry	Aboriginal and Torres Strait Islanders (First Australians)	DSM-5 diagnostic threshold eating disorders prevalence	Epidemiology study	Face to face interviews Logistic regression	Mental HRQoL, BMI, questions adapted from the EDE, ARFID questions. First Australian status. Physical HRQoL, demographics	92	53% F (mean = 36.49y)	First Australian	27% of First Australian respondents had an eating disorder, significantly higher than for other Australians. 1 respondent endorsed ARFID
*Hay * *(SA, Australia)* [7] *J Eat Disord*	Representative late adolescent/adult population sample	Assessing burden and HRQoL of EDs in Australian population	Epidemiology	Face-to-face survey interviews	Health Omnibus surveys [55]:(Demographics, DSM-5 ED features, functional impact on role performance, HRQoL, SF12v1	2014 = 2732 (9 ARFID) 2015 = 3005 (10 ARFID)	ARFID only: 2014 11% F 2015 50% F (≥ 15y, ARFID median = 46y)	Not stated	ARFID was reported in 1 in 300 people, was associated with poorer mental HRQoL (compared to non-ED participants) and significant functional impairments. Mean BMI was higher than for AN, but lower than no-ED
*Pinhas * *(Australia; Canada; UK)* [29] Int J Eat Disord	Representative child population sample	Can latent class analysis (LCA) classify ED symptoms in children that can be mapped onto DSM-5 diagnoses, and are these consistent between countries	Paediatrics	Latent class analysis of clinician case details from online survey	Clinician-completed questionnaires on cases (socio-demographic information, diagnosis, comorbidity, management, and short-term outcomes)	N = 436 (Australia n = 70) 26/70 who clustered as “more consistent with ARFID”	49% F (5-12y)	Not stated	LCA performed on eating symptoms clustered into 2-class model across all populations. Cluster 1 (74.6% of Australian sample) exhibited symptoms consistent with AN (DSM-IV TR). Cluster 2 was distinct from the AN group, and was more consistent with the DSM-5 category of ARFID
*Newport * *(New Zealand)* [56] Thesis	Representative child population sample (NZ birth cohort (Growing Up In New Zealand (GUINZ))	Exploring food intake and temperament of children aged 4.5y. Compared 'fussy eaters' vs. 'non-fussy' eaters	Epidemiology	Mother and Child Proxy Questionnaire completed in a face-to-face computer assisted personal interview	Socio-demographic data, child food intake (food frequency data (FFQ)), anthropometrics, Mother and Child Proxy Questionnaire (including child diet and nutrition, general health, motivation and emotion, and relationships)	6156	48.6% F (49–68 months (mean = 53.95y, SD = 1.55)	European (60%) Māori (13.4%), Pacific (13.2%), Asian (12.1%), and Middle Eastern, Latin American, or African (1.4%)	Prevalence of severe fussy eating was 1.9% (increased to 2.8% when starchy vegetables were classified as grain food group rather than vegetable). Predictors of fussy eating included gender, temperament dimensions (Attention to change, Fear) and socio-economic status. Increased fussy eating was observed in males, children with higher levels of fear, lower levels of attention to change, or who lived in more deprived households
*Claudino * (*Oceania; North America; South America, Asia; Africa; Europe) [52] BMC Med	Mental health professionals registered with WHO's Global Clinical Practice Network	Application of ICD-10 and ICD-11 diagnostic guidelines for FED to case vignettes – evaluate clinical utility of diagnostic guidelines	Psychiatry	Experimental vignette-based case-controlled	Diagnosis selection for vignettes, a set of questions related to clinical utility of the diagnostic guidelines (including ease of use, goodness of fit, and clarity)	2288 total (Western Pacific – Oceania = 66 (2.9%))	56% male (mean = 44.52y, SD = 10.91)	Not stated	Respondents rated ICD-11 favourably with use of the “Extremely” category, for ‘ease of use’ (85% or respondents), ‘goodness of fit’ (88%), and ‘clarity and understandability’ (88.6%). Clinicians diagnosed AN correctly using both ICD-10 and ICD-11, however the ARFID vignette (as per ICD-11) resulted in multiple diagnoses using ICD-10 criteria which reduced with ICD-11 criteria
*Jackson * *(New Zealand)* [47] Speech Lang Hear	Health professionals (speech-language therapist, dietitians and medical practitioners) working with children and feeding difficulties in New Zealand	Explored changes in perspective from 2013 to 2018 among health professional, regarding picky eating behaviour and the DSM-5 ARFID diagnosis	Speech-language therapy, Dietetics, General practice, paediatrics	Online survey, case vignette Qualitative content analysis	Respondent demographics, vignette diagnosis, open ended survey questions	141 total (2013 = 73, 2018 = 68)	Not stated	Not stated	There was a continued lack of consensus for diagnosing children with ARFID in 2018
*Le * *(South Australia, Australia)* [35] Psychol Med	Representative samples of individuals aged 15 + years living in South Australia	Present Health state utility values (HSUV) for a broad range on DSM-5 EDs	Epidemiology	Face to face interviews Multiple linear regression models	HRQoL was assessed using SF-12, EDE, ARFID-specific questions assessing the presence and reasons for current food avoidance/restriction based on DSM-5 criteria for ARFID	5609 total (ARFID = 13)	2232 M 3355 F (15 + y (EDs mean = 40y No-ED mean = 48.8y))	Not stated	HSUVs are used to determine quality-adjusted life years (or measure disease burden). The average HSUV was lowest in the ED threshold group (0.68, SD = 0.13), followed by the ARFID group (0.74, S.D. = 0.14) indicating high burden
*Selllbom * *(Dunedin, New Zealand)* [57] Assessment	University population sample (non-clinical)	Hierarchical Taxonomy of Psychopathology (HiTOP) measurement subgroup—Results from phase 1: Developing scales for the somatoform spectrum and eating disorders	Psychopathology taxonomy	Exploratory factor analysis In-person study measure completion	Selected scales from the MMPI-2-RF, EDDS-DSM-5	550	115 M 433 F 1 Transgender 1 unreported (17-51y (mean = 19.76)	NZ European (73%). Other European (17%), New Zealand Māori (10%), Chinese, Pacific Islander, Indian and “other” also represented	10- item scale for ARFID was developed. No large correlations between the ARFID scale and existing ED symptoms demonstrating discriminant validity of the scale. May help distinguish between non-weight phobic AN, and ARFID

12-Item Short Form Health Survey (SF-12), Anorexia nervosa (AN), Avoidant/Restrictive Food Intake Disorder (ARFID), BMC medicine (BMC Med), Diagnostic and Statistical Manual of Mental Disorders, 4th Edition, Text Revision (DSM-IV-TR), The Diagnostic and Statistical Manual of Mental Disorders, Fifth Edition (DSM-5), Eating disorder (ED), Eating Disorder Diagnostic Scale (EDDS), The Eating Disorder Examination (EDE), Food Frequency Questionnaire (FFQ), Health state utility value (HSUV), Health-Related Quality of Life (HRQOL), Journal of eating disorders (J Eat Disord), Journal of Gastroenterology and Hepatology (J Gastroenterol Hepatol), Kilogram per square metre (kg/m2), Latent class analysis (LCA), Minnesota Multiphasic Personality Inventory (MMPI), Psychological Medicine (Psychol Med), Speech, Language and Hearing (Speech Lang Hear), The International Classification of Diseases 10^th^ revision (ICD-10), The International Classification of Diseases 11^th^ revision (ICD-11), The International journal of eating disorders (Int J Eat Disord), World Health Organization (WHO)

^*^ Indicates region not country was reported

**Table 4:** Guidelines

Author and year (Country)	Population focus	Study focus	Summary results
Hay (Australia, New Zealand) [53] Aust N Z J Psychiatry	ED	Treatment Guidelines	No specific treatment is recommended for ARFID as there are no trials to guide practice
Ralph (Australia) [30] J Eat Disord	EDs in people of higher weight	Clinical practice guidelines	People with higher weight should be assessed and managed for ARFID in the same way as people with lower weight. The NIAS, and CFNS are possible measures for adults and children respectively. No treatment recommendation for ARFID, but CBT noted as promising

Avoidant/Restrictive Food Intake Disorder (ARFID), Eating disorder (ED), Child’s Food Neophobia Scale (CFNS), Cognitive behavioural therapy (CBT), The Australian and New Zealand journal of psychiatry (Aust N Z J Psychiatry), Journal of eating disorders (J Eat Disord)

### Synthesis of results

See Table 5.

**Table 5:** Summary of key data extracted from sources included in this systematic review

	Case reports	Treatment interventions	Cross-sectional studies	
	(Clinical)	(Clinical)	(Clinical)	(Non-Clinical)
Number of Studies	4	15	1	8
Sample (n) range	1	1–32	10	66–6156
Age range (y)	11–64	2–42	> 16	5-Adulthood*
Gender	50% F	28% F	100% F	44% F^
Engagement method	Clinical assessment	In-home Intervention Telehealth Face to face clinic sessions	Retrospective clinical data collation	Face-to-face interviews Face-to-face computer assisted personal interview Questionnaires Online survey Vignette-based online survey In person study measure completion
Assessment Instruments	Montréal Cognitive Assessment DSM-5 ED assessment	MINI EDE-Q DASS-21	–	SF12v1 FFQ EDE MMPI-2-RF EDDS-DSM-5
Other measures	Nutritional blood screen MRI CT scan DEXA bone scan Neurological status Neuropsychology examination BMI X-ray Physical examination Dietary history Background history	BMI Measures relating to food consumption and treatment acceptability Caregiver satisfaction	Diagnosis Demographics Relapse/readmission rates Length of stay BMI Refeeding method Medical complications	ARFID-related questions Demographics Physical HRQoL Diagnosis Comorbidity Management Short-term outcomes Anthropometrics Background history
Key Findings	Health consequences of ARFID can be varied, severe, and irreversible if not addressed.	Interventions implemented included: CBT Behaviour analytic treatment FBT + UP-C/A Caregivers prioritised quick and effective treatments over minimising side-effects. Behaviour analytic treatments had high caregiver acceptability ratings.	Only one study reported clinical cases, and these were adults. Clinical cross-sectional studies are urgently required.	Clinicians rated ICD-11 favourably. An ARFID vignette resulted in multiple diagnoses under the ICD-10 condition. When presented with a typical case vignette suggestive of ARFID, the majority of NZ health professional respondents did not label the case as ARFID in a multichoice answer, and 89.7% said there was “no consensus” on a label. ARFID prevalence in South Australians was 1 in 300, and is associated with poorer mental HRQoL and significant functional impairments (compared to those without an ED). HSUV for individuals with ARFID is low (0.74), secondary only to those with threshold ED (0.68). Prevalence of severe fussy eating in NZ children = 1.9–2.8%.

^*^ Ages not reported in one study of health professionals, and > 15y or  > 16y age range described in two other studies

^ Gender was not reported for one cross sectional study of health professionals, and a further study reported a single transgender individual, and one individual of undisclosed gender that are not included in the % F reported in the summary table

Avoidant/Restrictive Food Intake Disorder (ARFID), Body mass index (BMI), Cognitive behavioural therapy (CBT), Computerised Tomography (CT), Depression Anxiety and Stress Scale 21 (DASS-21), Dual X-ray Absorptiometry (DEXA), The Diagnostic and Statistical Manual of Mental Disorders, Fifth Edition (DSM-5), Eating disorder (ED), Eating Disorder Diagnostic Scale (EDDS), The Eating Disorder Examination (EDE), Eating Disorder examination questionnaire (EDE-Q), Family Based Treatment (FBT), Female (F), Food Frequency Questionnaire (FFQ), Health-Related Quality of Life (HRQOL), Health state utility values (HSUVs), The International Classification of Diseases 10th revision (ICD-10), The International Classification of Diseases 11th revision (ICD-11), Mini International Neuropsychiatric Interview (MINI), Minnesota Multiphasic Personality Inventory (MMPI), Magnetic Resonance Imaging (MRI), 12-Item Short Form Health Survey (SF-12), Unified Protocols for Transdiagnostic Treatment of Emotional Disorders in Children and Adolescents (UP-C/A)

## Discussion

### Summary of findings

This review was developed around the research question, “To date, what stakeholders, methodologies, and results have been reported by studies examining ARFID within Australasia?”

This review covered a wide range of studies examining ARFID in clinical and non-clinical populations, as well as healthcare professionals' and caregivers' perspectives, using diverse study types and methodologies. This review reported on the population focus, methodologies, and significant findings of each study which are addressed in respective sections below.

### Population focus

#### Individuals with lived experience

People with ARFID are at risk for significant and irreversible damage secondary to malnutrition, emphasizing the importance of timely diagnosis and intervention. Failure to adequately treat ARFID can lead to serious health consequences, including delayed growth and development, organ damage, and in severe cases, death [28, 36, 39, 41].

In addition to physical health consequences, people who experience ARFID may also face lower mental health-related quality of life and significant functional impairment. ARFID can have a profound impact on social and emotional wellbeing, as well as daily functioning, including school or work participation [7, 35].

The present scoping review found that sex distribution in ARFID studies was comparatively balanced, with both male and female participants represented in the literature. This is notable given the tendency of eating disorder research to focus on female participants, and is likely a reflection of the more balanced prevalence of ARFID in both sexes [7]. In contrast, data on gender were less frequently reported, and there was no literature included that presented data on sexual minorities and LGBTQIA + individuals. This is concerning given that these individuals may be at increased risk of eating disorders [59], yet this population is not addressed in the current Australasian ARFID literature.

The review found a lack of ethnicity data in many studies (14 sources), with non-European populations frequently underrepresented (or grouped with other ethnicities) when they were reported [38, 50, 51]. The research that reported Indigenous groups was limited to just three publications, two from NZ [56, 57] and one from Australia [34], with Māori and First Australian samples respectively. Excluding Indigenous populations, such as Māori, Pasifika, and Aboriginal and Torres Strait Islanders, from research has detrimental effects, perpetuating health inequalities and hindering effective interventions in these communities. It also limits understanding of their unique experiences and cultural perspectives, including food practices and beliefs [60, 61], impeding progress towards health equity.

Caregivers of individuals with ARFID expressed a preference for effective treatment (over minimizing side effects) [51] which is important to consider in the management of this condition. However, the family or caregiver dynamic may also benefit from direct intervention, highlighting the need to consider the complex environmental factors that can impact ARFID management. For example, caregivers may have conflicting beliefs or expectations about treatment, leading to disagreements or resistance to certain interventions [28]. There was no Australasian data that addressed caregivers’ own behaviours and attitudes about food.

### Healthcare professionals

Healthcare professionals involved in the diagnosis and management of ARFID in Australasia from these sources were from a diverse range of professions, such as paediatricians, gastroenterologists, dietitians, mental health specialists, and speech and language therapists. However, despite the growing recognition of ARFID as a distinct feeding and eating disorder among specialists, it is clear that many healthcare professionals remain unfamiliar with the diagnosis [47] and may be unlikely to have the necessary skills and knowledge to manage the condition effectively. This is particularly concerning given the diagnostic inconsistency that exists, and the overlap of ARFID with paediatric feeding disorder (PFD).

**Fig. 2:**
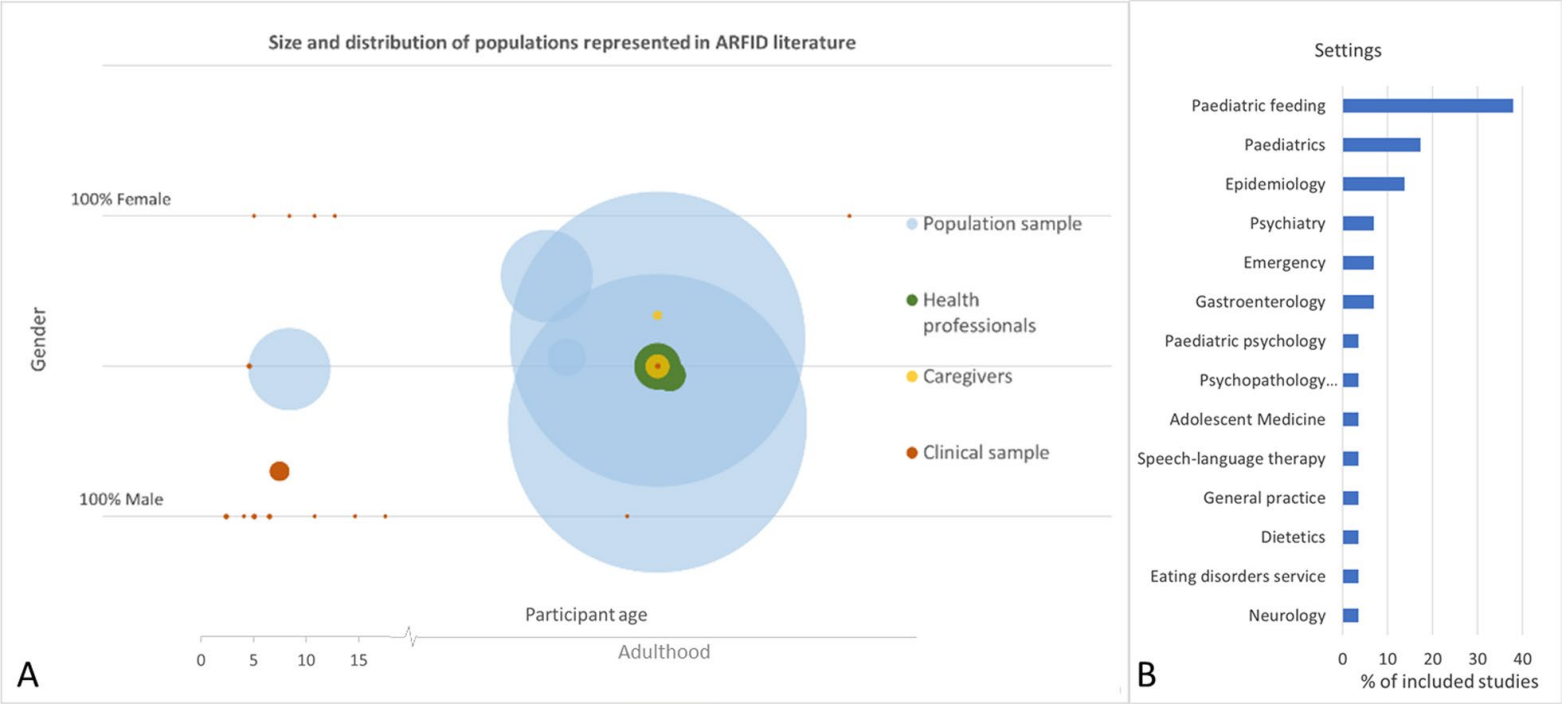
(A) A graphic representation of the demographic characteristics (gender, age (< 15years in years, and > 15 grouped in Adulthood range*) and sample sizes of various study population focuses. Each bubble represents a specific population group, differentiated by colour. The size of each bubble corresponds to the sample size. *Note: age of participants was not reported, or reported as > 15 years in 5 studies of adults. Therefore, studies of adult populations have been grouped on the x axis into an “adulthood” age range; (B) Presentation/treatment setting for ARFID reported within the studies included in this review, ordered by decreasing frequency (measured by percentage of included studies)

The complexity of proposed diagnostic subgroups, medical and nutritional complications, and potential comorbidities (that may include neurodevelopmental) means that ARFID is treated well by multidisciplinary teams (MDT)s [41]. The wide range of presentation settings represented in Fig. 2B, which includes various healthcare specialties involved in the research study or patient treatment, also emphasises the need for widespread shared understanding of the disorder across the healthcare spectrum.

### Methodologies

The majority of studies (24/30) on ARFID presented in this scoping review relied on quantitative data, with a heavy emphasis on self-reported measures. Utilizing self-reported surveys with scales allows for a standardized measurement of symptoms. However, it is important to note that such quantitative data obtained from self-reported surveys primarily provides a retrospective overview of symptoms and may also be subject to potential biases inherent in self-reporting. It may not capture the in-depth personal experiences of individuals, including how these symptoms impact them, their families, or their overall quality of life.

Most interventions for ARFID were described in single case reports rather than rigorous study designs. This highlights the need for more comprehensive research on interventions for ARFID, including controlled trials with larger sample sizes. Suitable powered treatment trials are essential for delivering data on treatment efficacy, and informing treatment guidelines which are currently absent for ARFID.

No longitudinal studies with a direct focus on ARFID met inclusion for the review. Long-term local research is needed to track the progression of ARFID and evaluate the effectiveness of interventions, particularly beyond three years (the longest follow up period in this review). This is particularly important given the potential for significant and irreversible damage associated with untreated ARFID. Longitudinal data collection faces a significant challenge due to the limitations of existing ARFID assessment measures, which are tailored to identifying the presence of a current ED rather than to capture prior eating disorder experiences. For instance, the PARDI questionnaire enquires about behaviours within the past 1–3 months, and the NIAS primarily focuses on current behaviours. Consequently, when relying on these current measures, there is a risk of overlooking an individual's past history of ARFID if they have previously recovered or transitioned to another eating disorder. We subsequently have very little data on the development course of ARFID, and the frequency with which individuals with ARFID may cross over to other ED presentations in the lifetime. This should be a focus for future research, particularly in light of the community identifying the “lifetime course of ARFID” as a key research priority in NZ (unpublished data).

Additionally, the lack of large clinical samples in the existing literature is a notable gap in knowledge. Research with larger samples would allow for a more comprehensive understanding of the condition, including its prevalence, presentation, and treatment outcomes.

The collection and presentation of biological data in the reviewed studies were limited to reports of severe ARFID presentations [28, 36, 39] or clinical in-patients populations [41]. By primarily studying clinical populations, the biological ranges in individuals with ARFID outside of clinical settings are not fully represented. Future research should aim to include a broader range of individuals to gain more comprehensive data in Australasians with ARFID.

### Significant findings

Although limited, the three month prevalence estimates of ARFID in an adult Australian population (0.3%) and prevalence of severe fussy eating in a NZ child birth cohort (1.9%) are comparable to the wide range of international prevalence estimates of ARFID in non-clinical populations (0.5–1.3% in children [3, 62], and 4.1–4.8% in adults [10, 63]).

The included literature presented both quantitative and qualitative results of interventions for ARFID, with a strong focus on behaviour analytics (e.g., exposure-based therapy, and contingent access) with fewer reports of FBT/CBT [27, 58]. The interventions described in the included sources ranged from individual and family-based therapies to group-based treatments, and often involved a multidisciplinary approach. However, the lack of large-scale clinical trials and longitudinal studies limits the generalizability and long-term effectiveness of these interventions. It is noted that a wealth of research exists of interventions performed in PFD [40], and the effectiveness of these approaches in treating ARFID should be further assessed with larger studies.

Population prevalence and Health-Related Quality of Life (HRQoL) data, highlighted the significant impact of ARFID on individuals and their families. Mental HRQoL is low and disease burden (measured by health state utility values (HSUV)) is high in individuals with ARFID, compared to non-ED populations [35, 64, 65]. This is consistent with poorer psychosocial health and school functioning scales reported in ARFID compared to healthy controls [17].

Research involving clinicians focused on the application of diagnostic manuals, such as ICD-10 vs ICD-11, and on the familiarity and confidence in diagnosing ARFID [47, 52]. Low confidence and familiarity within surveyed Australasian clinicians mirrored international findings [25] that much work needs to be done in this area. The inclusion of ARFID in the International Classification of Diseases 11th revision (ICD-11) is a significant step forward in the recognition and visibility of this disorder, and may help distinguish ARFID [52] in settings that rely on ICD diagnoses rather than DSM.

The 2020 systematic scoping review by Bourne et al. identified four key areas of focus for the next five years in ARFID research: characterizing the drivers of food avoidance/restriction, validating reliable assessment tools, collecting epidemiological data, and expanding research beyond feeding or eating disorders (such as psychobiology of appetite and ARFID psychiatric comorbidity). While some progress has been made in addressing these areas, many recommendations from Bourne et al*.* have not been implemented in subsequent studies. For instance, emerging instruments like PARDI and EDE-ARFID, which were deemed suitable for ARFID assessment and data comparability, remained unused in the studies reviewed here. Additionally, there is a lack of updated epidemiological data in Australasia regarding non-clinical adult ARFID prevalence, with the last estimate of 0.3% by Hay et al*.* in 2017 [31], which was incorporated in the previous review, remaining unchanged.

### Limitations

The present study has some limitations that must be acknowledged. Despite the development of a limited number of standardized ARFID measures (Nine Item ARFID Screener (NIAS)(2018)[66, 67]; Pica, ARFID, Rumination Disorder Interview (PARDI)(2019)[68], and Pica, ARFID and Rumination Disorder Interview ARFID Questionnaire (PARDI-AR-Q)(2022), none of these measures were utilised by the studies included in this review. For several cross-sectional studies, the dataset was collected prior to (or soon after) the publication of these measures. Other studies had a broader ED focus, and therefore used the EDE or EDE-Q. This limits the comparability of findings, as ARFID has been defined or assessed in a range of ways (e.g., a clinical diagnosis in a case study, versus assessment based on one or two self-report answers in a population study). Therefore, quantitative measures may be highly dependent on the specificity and sensitivity of the assessment method employed in the sources.

Secondly, the continuing use of distinct but overlapping diagnostic labels in the literature, particularly paediatric feeding disorder (PFD), means that relevant literature may have been missed. This could limit the comprehensiveness of our review and conclusions. In particular, the OVID database search required limiting the search terms "ARFID" or "Avoidant restrictive food intake disorder" to the abstract field to ensure a manageable number of relevant results. This strategy may miss PFD literature where an ARFID diagnosis was also present, if not mentioned in the abstract text. This situation is apparent in several case reports by Taylor et al*.* where PFD is the diagnosis used in the title and abstract, but “ARFID” " or "Avoidant restrictive food intake disorder" was used as a key word, prompting follow up with the author who confirmed a diagnosis of ARFID was also made. This particular issue affects sources that would have been identified only by an OVID database search and not present in any of the other searched databases which is likely to be low in number. However, use of other terms where ARFID is not described explicitly in the source is a more general issue.

The under-representation of Indigenous populations in studies examining ARFID is a notable limitation in the existing literature. This lack of representation is concerning, and relates to how Indigenous cultures experience sociocultural disadvantage affecting access to treatment for eating disorders (for example, see Lacey et al., 2020 [69]), and participation in research [70]. Thus, the generalizability of findings on ARFID may be limited, and future research in this area should prioritize including diverse populations, including Indigenous peoples.

Finally search strategies for university theses are not well established. The limited number of University repositories in NZ could be hand searched, however this was not realistic for the far greater number of Australian Universities. Using Trove—National Library of Australia to search for theses from a large number of Australian Universities was an efficient option, but we acknowledge that not all Australian Universities may have made their thesis repositories available through this service. Additionally, thesis repositories may be incomplete, as in the case of the NZ thesis included in this review which was identified instead through personal communication.

### Recommendations

This scoping review on ARFID in Australasia has identified several gaps in the current local literature and offers recommendations for future research in this area.

- Research on larger-scale clinical samples is necessary to validate existing studies and to provide more accurate estimates of the prevalence of ARFID in Australasia. Diverse participant samples should be included, such as individuals of different ages, genders, and ethnicities. It is important to be aware of any gender biases in clinical populations, especially in studies conducted in eating disorder clinics, which may predominantly see women.
- Population-based studies are needed to accurately estimate the prevalence of ARFID in Australasia during childhood and adulthood.
- Further research is necessary on ARFID in Indigenous populations as they may be at higher risk. Understanding unique risk factors and appropriate interventions for this population is essential.
- For studies investigating children with feeding or eating difficulties, it is important to indicate if an ARFID diagnosis is met, even in the context of paediatric feeding disorder. This can help ensure that results and findings are relevant to ARFID and can inform future research and interventions.
- Further research is necessary to determine the effectiveness of treatment interventions in ARFID populations. The PFD literature is a valuable resource for behaviour interventions that may be effective or adaptable in ARFID populations, and researchers should consider using these interventions when designing ARFID studies.
- Utilisation of standardized measures for ARFID screening and diagnosis in future studies is imperative to facilitate comparability of findings.

Funders play a critical role in supporting research that can improve our understanding of ARFID and inform effective interventions. Here are some recommendations for funders to consider when supporting ARFID research:

- Provide appropriate funding to support the inclusion of children in research studies. ARFID is a condition that often affects children, and research that includes children can provide valuable insights into the nature of ARFID and how it can be treated. However, studies involving children can be more complex and require additional resources. Funders should be prepared to provide adequate funding to support the inclusion of children in ARFID research.
- Support research that reaches "hard to reach" populations, such as Indigenous populations, and encourages community engagement [70]. Indigenous populations may be at higher risk for ARFID, but they may also face additional barriers to accessing healthcare and participating in research studies. Funders should support research that is designed to reach these populations and encourage community engagement through culturally appropriate means, such as yarning methodology or hui. This will help ensure that research is inclusive and representative of the population being studied.
- Support involvement of people with lived experience of ARFID to be involved co-production at all stages of the research process.
- Fund research into ARFID awareness and education for healthcare professionals, caregivers, and family members. ARFID is a relatively new diagnosis, and a need for increased awareness and education among healthcare professionals, caregivers, and family members is evident. Local research like this can inform the development of educational resources and training programs that can improve care for individuals with ARFID and raise awareness of the condition.

## Conclusions

In conclusion, while this review serves as a valuable starting point for ARFID research in Australasia, there are critical areas that require immediate attention. These include obtaining more accurate population prevalence estimates of ARFID in children, conducting larger-scale studies in clinical populations using well-validated standardized measures, and enhancing education and training for healthcare professionals to improve diagnosis and management of ARFID. Additionally, controlled trials for ARFID interventions, such as behaviour analytics, should be considered, along with longitudinal studies that track the progression of ARFID over time and shed light on the stability of the diagnosis and the risk of developing other eating disorders throughout the lifespan. Urgent research is needed to enhance our understanding of ARFID in Australasia, to ultimately improve outcomes for affected individuals.

## Data Availability

Not applicable.

## Abbreviations

ANZCTR: Australian New Zealand Clinical Trials Registry
APA: American Psychological Association
ARFID: Avoidant/Restrictive Food Intake Disorder
ASD: Autism Spectrum Disorder
BMI: Body Mass Index
CBT: Cognitive-Behavioural Therapy
DSM: Diagnostic and Statistical Manual of Mental Disorders
DSM-5: Diagnostic and Statistical Manual of Mental Disorders, 5th edition
EDE: Eating Disorders Examination
EDE-Q: Eating Disorders Examination Questionnaire
ED: Eating Disorder
EMBASE: Biomedical and pharmacological database
FBT: Family-Based Therapy
HSUV: Health State Utility Values
ICD-10: International Classification of Diseases, 10th Revision
ICD-11: International Classification of Diseases, 11th Revision
LGBTQIA +: Lesbian, Gay, Bisexual, Transgender, Queer/Questioning, Intersex, Asexual, and other related identities
MDT: Multidisciplinary Team
MRI: Magnetic Resonance Imaging
NZ: New Zealand
OVID: Online database platform
PFD: Paediatric Feeding Disorder
PRISMA: Preferred Reporting Items for Systematic Reviews and Meta-Analyses
